# Genomic surveillance of SARS‐CoV‐2 strains circulating in Iran during six waves of the pandemic

**DOI:** 10.1111/irv.13135

**Published:** 2023-04-16

**Authors:** Kaveh Sadeghi, Sevrin Zadheidar, Arghavan Zebardast, Ahmad Nejati, Marziyeh Faraji, Nastaran Ghavami, Shirin Kalantari, Vahid Salimi, Jila Yavarian, Adel Abedi, Nazanin Zahra Shafiei Jandaghi, Talat Mokhtari‐Azad

**Affiliations:** ^1^ Virology Department, School of Public Health Tehran University of Medical Sciences Tehran Iran; ^2^ Research Center for Antibiotic Stewardship & Antimicrobial Resistance Tehran University of Medical Sciences Tehran Iran; ^3^ Mathematics Department Shahid Beheshti University Tehran Iran

**Keywords:** Iran, NGS, SARS‐CoV‐2, variants

## Abstract

**Background:**

SARS‐CoV‐2 genomic surveillance is necessary for the detection, monitoring, and evaluation of virus variants, which can have increased transmissibility, disease severity, or other adverse effects. We sequenced 330 SARS‐CoV‐2 genomes during the sixth wave of the COVID pandemic in Iran and compared them with five previous waves, for identifying SARS‐CoV‐2 variants, the genomic behavior of the virus, and understanding its characteristics.

**Methods:**

After viral RNA extraction from clinical samples collected during the COVID‐19 pandemic, next generation sequencing was performed using the Nextseq and Nanopore platforms. The sequencing data were analyzed and compared with reference sequences.

**Results:**

In Iran during the first wave, V and L clades were detected. The second wave was recognized by G, GH, and GR clades. Circulating clades during the third wave were GH and GR. In the fourth wave, GRY (alpha variant), GK (delta variant), and one GH clade (beta variant) were detected. All viruses in the fifth wave were in GK clade (delta variant). In the sixth wave, Omicron variant (GRA clade) was circulating.

**Conclusions:**

Genome sequencing, a key strategy in genomic surveillance systems, helps to detect and monitor the prevalence of SARS‐CoV‐2 variants, monitor the viral evolution of SARS‐CoV‐2, identify new variants for disease prevention, control, and treatment, and also provide information for and conduct public health measures in this area. With this system, Iran could be ready for surveillance of other respiratory virus diseases besides influenza and SARS‐CoV‐2.

## 1 INTRODUCTION

A highly transmissible and pathogenic CoV, severe acute respiratory syndrome coronavirus 2 (SARS‐CoV‐2), emerged in late 2019 and has caused a pandemic of acute respiratory disease known as “coronavirus disease 2019” (COVID‐19).^1^

As of March 16, 2023, 760 360 956 cases of SARS‐CoV‐2 with 6 873 477 deaths were reported worldwide, and in Iran from January 3, 2020, to March 16, 2023, there have been 7 575 927 confirmed cases of COVID‐19 with 144 993 deaths.^2^ The first confirmed cases from Iran were reported in Qom province on February 19, 2020.^3^ On July 30, 2020, scientists from Tehran University of Medical Sciences and officials from Iran's Center for Infectious Disease Control, MoHME, released the clinical and virological characteristics of the first seven cases of COVID‐19 in Iran.^3^ Clades L (reference sequence) and V (NSP6‐L37F and NS3‐G251V) were discovered in Iran during the first wave from February to May 2020. The second wave in late June until September 2020 was recognized by G, GH, and GR clades. The clades that circulated in the third wave from October to December 2020 were GH and GR. In the fourth wave from the beginning of April to June 2021, GRY (alpha variant) and GK (delta variant) were detected. All viruses of the fifth wave from August to October 2021 belonged to the GK clade (delta variant).

Coronaviruses have polycistronic genomes (ranging from 26.0 to 32.0 kb), and approximately two thirds of their genomes are occupied by two large ORFs (ORF1a and ORF1b) that encode 16 nonstructural proteins (NSP1‐NSP16), and one third of them encode structural proteins, spike (S), envelope (E), membrane (M), and nucleocapsid (N), and accessory proteins.^4^, ^5^

The genome additionally consists of 11 open reading frames (ORFs) (ORF3a, ORF3b, ORF3c, ORF3d, ORF6, ORF7a, ORF7b, ORF8, ORF9b, ORF9c, and ORF10), which encode accessory proteins. They are not vital for viral replication; however, they are involved in pathogenesis.^6^, ^7^

Coronaviruses are biologically diverse and mutate rapidly.^8^ The virus's properties are largely unaffected by most of changes. However, some changes may have an impact on the properties of the virus, such as how easily it spreads, the severity of the disease it causes, or how well vaccines, therapeutic medicines, diagnostic tools, or other social and public health measures work.^9^ Since the beginning of the COVID‐19 pandemic, different genetic lineages of SARS‐CoV‐2 have emerged and spread worldwide.^9^ The SARS‐CoV‐2 variants that may pose an increased risk to public health have been divided into the following three groups by WHO: Variants under monitoring (VUM), variants of interest (VOIs), and variants of concern (VOCs). A variant with genetic changes that are thought to affect the characteristics of the virus is called a VUM, and there are some indications that it may pose a threat to public health and safety in the future. Variants that have been found to cause community spread in multiple cases, clusters, or countries are defined as VOIs. The definition of a VOC is an increase in transmissibility and virulence or a decrease in the efficacy of current public health, social, and therapeutic measures.^10^ There are currently 11 clades in the GISAID nomenclature system, which is based on shared marker mutations. The L and S clades formed early in the pandemic before the L split into V and G. The GR, GH, GV, and GK clades split from base clade G. GR evolved into GRY, which later developed into GRA, the current dominant clade. The O clade contains all sequences that have not been classified.^11^

The gold standard for monitoring and identifying new variants in SARS‐CoV‐2 is whole genome sequencing (WGS) using next generation sequencing (NGS). All SARS‐CoV‐2 genes can be sequenced using this method, including those encoding non‐structural proteins and other regions.^12^ It is essential to maintain constant monitoring of the genetic diversity of SARS‐CoV‐2 in order to (a) ensure that vaccines and immune‐based diagnostic or therapeutic interventions are effective, (b) offer a treatment that is much more stable, and (c) observe the pattern of the virus's geographic spread during the ongoing pandemic.^13^, ^14^

## 2 METHODS

### 2.1 Sample collection

This study was a population‐based cross‐sectional assessment of data on 330 throat swabs of COVID‐19 confirmed cases from all over Iran during the sixth wave of COVID‐19, which were sent to the National Influenza Center (NIC), Department of Virology, School of Public Health, Tehran University of Medical Sciences, Iran, for whole genome sequencing of SARS‐CoV‐2 strains. These samples included inpatients and outpatients from health centers and hospitals across the country. They were chosen randomly following primary detection by Real‐time PCR based on having the ct values of below 25.

### 2.2 NGS

Viral RNA was extracted using the High Pure Viral Nucleic Acid kit (Roche, Germany). For library preparation and NGS performance, two platforms were used: Illumina and Oxford Nanopore Technologies. For Illumina platform, the Nextera DNA Flex kit (Illumina, USA) and the Respiratory Virus Oligo Panel kit (Illumina, USA) were used for library preparation and hybridization. After clean‐up, quality control evaluation was done using Qubit (Thermo Fisher, USA). At last, sequencing was performed utilizing NextSeq 550 machine (Illumina, USA).

For Nanopore platform, PCR tiling of SARS‐CoV‐2 virus with rapid barcoding and Midnight RT‐ PCR Expansion (SQK‐RBK110.96 and EXP‐MRT001) (Version: MRT_9127_v110_revM_14Jul2021) was used. The library was prepared by the Rapid Barcoding Sequencing (SQK‐RBK004) kit (Version: RBK_9054_v2_revX_14Aug2019) from Oxford Nanopore Technologies®, UK. The library concentration was measured using the Qubit HS dsDNA assay kit (Thermo Fisher, USA). Finally, the sequencing was done by the GridION with the R9.4.1 flow cells (Oxford Nanopore Technologies®, UK).

### 2.3 Data analysis

All the reads were mapped to the SARS‐CoV2 reference genome assembly for data investigation. The assembled viral genome was of high quality and contained no unknown nucleotides. The gathered genomes were studied by CoVsurver mutations Application in GISAID and aligned using the sequence alignment program BioEdit. Finally, all sequences were submitted in GISAID.

## 3 RESULTS

In this study, 330 COVID‐19 confirmed cases from the sixth wave of COVID‐19 in Iran were subjected to NGS. These specimens were oropharyngeal swabs collected from all over the country. The period of sample collection was from March 6, 2022, to March 20, 2022. Out of 330 samples, 143 (43.3%) were female and 187 (56.7%) were male. The age ranged from younger than under 1‐year‐old to 113 years old. All of evaluated SARS‐CoV‐2 strains in the sixth wave were Omicron, in which, out of all 330 strains, 153 (46.3%) were BA.1, 159 (48.2%) were BA.2 and 18 (5.5%) were mixed lineage (ML). We analyzed amino acid (aa) substitutions, insertions and deletions of each protein separately compared to hCoV‐19/Wuhan/WIV04/2019 in GISAID. Then SARS‐CoV‐2 variants and amino acids changes in structural, non‐structural and accessory proteins were compared with SARS‐CoV‐2 strains circulated in Iran during the first five waves which were evaluated in our previous study.^3^, ^15^

Amino acids changes in structural proteins were listed in Table 1 and those related to nonstructural proteins were mentioned in Table 2. It should be noted that amino acid substitutions in accessory proteins were detected in a limited number of strains in the sixth wave. The highest rate of substitution in these proteins was 1.3% among BA.1 and BA.2 variants as follows: 1.3% of BA.1 strains had NS7a‐P99S substitution. Besides, 1.3% of BA.2 strains had NS3‐H78Y substitution.

**TABLE 1:** Amino acid changes detected in structural proteins of SARS‐CoV2 strains circulated in the sixth wave of the pandemic which were detected in more than 70% of each variant and compared to shared aa changes during the first five waves in Iran.

Genes		6th wave			Shared changes with previous waves				
		BA.1	BA.2	ML	1st	2nd	3rd	4th	5th
Structural proteins	S	D614G D796Y E484A G142D G339D H655Y K417N N440K N501Y N679K N764K N856K N969K P681H Q493R Q498R Q954H S371L S373P S375F S477N T478K T547K Y505H	D405N D614G D796Y E484A G142D G339D H655Y K417N N440K N501Y N679K N969K P681H Q493R Q498R Q954H R408S S373P S375F S477N T376A T478K V213G Y505H P26DEL P25DEL A27S L24DEL	D614G D796Y E484A G142D G339D H655Y K417N N440K N501Y N679K N969K P681H Q493R Q498R Q954H S371L S373P S375F S477N T478K T547K Y505H P26DEL P25DEL A27S L24DEL		D614G	D614G	D614G N501Y P681H	D614G T478K G142D
	E	T9I	T9I	T9I					
	M	A63T D3G Q19E	A63T Q19E	A63T D3G Q19E					
	N	R203K G204R	R203K G204R	R203K G204R					

**TABLE 2:** Amino acid changes detected in non‐structural proteins of SARS‐CoV2 strains circulated in the sixth wave of the pandemic which were detected in more than 70% of each variant and compared to shared aa changes during the first five waves in Iran.

Genes	6th wave			Shared changes with previous waves				
	BA.1	BA.2	ML	1st	2nd	3rd	4th	5th
Nonstructural	NSP3‐A1892T NSP3‐K38R NSP3‐L1266I NSP3‐S1265del NSP4‐T492I NSP5‐P132H NSP6‐G107del NSP6‐S106del NSP6‐I189V	NSP3‐K38R NSP3‐G489S NSP4‐T492I NSP5‐P132H NSP6‐G107del NSP6‐S106del NSP12‐P323L	NSP3‐A1892T NSP3‐K38R NSP3‐L1266I NSP3‐S1265del NSP4‐T492I NSP5‐P132H NSP6‐G107del NSP6‐S106del NSP6‐I189V	NSP12‐P323L	NSP12‐P323L	NSP12‐P323L	NSP4‐T492I NSP12‐P323L	NSP4‐T492I NSP12‐P323L

## 4 DISCUSSION

As an RNA virus, SARS‐CoV‐2 has a high rate of mutations, resulting in ongoing evolution over time that could affect replication, infectivity, transmissibility, virulence, and immunogenicity.^16^ Increasing transmissibility, pathogenicity, and the capacity to evade natural or vaccine‐induced immunity are all potential outcomes of emerging variants.^17^ Analysis of whole‐genome sequences is essential for monitoring its increased transmissibility and virulence‐altering potential. In this study, we reported the circulation of distinct lineages of SARS‐CoV‐2 Omicron variant during the sixth wave in Iran and its comparison with the previous waves. Besides, aa changes related to transmissibility, infectiousness, and pathogenicity in SARS‐CoV‐2 strains circulated during six waves of COVID‐19 in Iran are discussed.

Since the development of the SARS‐CoV‐2 infection in December 2019, a few VOCs have arisen and quickly spread with a worldwide circulation.^18^ Alpha (B.1.1.7), Beta (B.1.351), Gamma (P.1), and Delta (B.1.617.2) are the previous four VOCs. The fifth VOC on November 26, 2021, was named Omicron (B.1.1.529), which prompted worldwide concern.^19^ On November 24, 2021, the variant B.1.1.529 was first reported to WHO by South Africa.^9^ The large number of mutations in Omicron was the most alarming feature.^20^ At first, the variant was a family of three rather than a single strain: BA.1, BA.2, and BA.3^21^, ^22^; later, BA.4 and BA.5, as well as numerous sublineages within BA.1 and BA.2, have been identified.^23^ Not long ago, WHO indicated that the rise of some Omicron variants, particularly XBB and its sublineages (shown as XBB*) and BQ.1 and its sublineages (shown as BQ.1*), would affect worldwide public health. From March 3, 2023, the ECDC removed BA.2, BA.4, and BA.5 from its list of SARS‐CoV‐2 VOCs, because these parental lineages are not circulating. WHO also updated its tracking system and operational definitions of VOCs, VOIs, and monitored variants, in March 2023.^2^ Our results in previous study showed that in Iran the V clade was discovered during the first wave. Clade V was identified by NSP6‐L37F in addition to NS3‐G251V. The second wave was recognized by groups G, GH, GR, and V clade (in one sample). G clade contained D614G mutation. Clade GR was characterized by S‐D614G and N‐G204R, while clade GH had substitutions at S‐D614G and NS3‐Q57H. The third wave circulating clades were GH and GR. The GH (beta), GRY (alpha), and GK (delta) clades were discovered during the fourth wave. S‐H69del, S‐V70del, S‐Y144del, and S‐N501Y, as well as S‐D614G and N‐G204R, made up the GRY clade (alpha). All viruses in wave 5 belonged to clade GK (S‐D614G and S‐T478K).^15^ In this study, we found that during the sixth wave of COVID‐19 pandemic in Iran, BA.1 and BA.2 lineages of Omicron variant were circulating. Besides, one of the distinct characteristics of this wave was discovering the mixed lineage (BA.1 + BA.2) in our samples.

The Omicron subvariants have shown notable changes regarding S protein mutants, especially in the N‐terminal area and the receptor‐binding domain, which are known to contain important neutralizing antibody epitopes.^24^ The spike protein of the first dominant SARS‐CoV‐2 Omicron variant, BA.1, contains 35 mutations from the initial SARS‐CoV‐2 variant that emerged in late 2019. BA.1 quickly became the most common variant worldwide after being discovered, and it has since developed into several other lineages.^23^ The spike protein of BA.1 has more than 30 mutations that make it less sensitive to vaccine‐induced antibody neutralization^25^; these substitutions are as follows: 375F, K417N, T547K, L981F, R346K.^23^, ^26^, ^27^, ^28^ K417N significantly reduces the ability of antigenic peptides to be loaded onto relevant human leukocyte antigen or HLA‐A class I, thereby eliminating host CD8+ T‐cell responses.^29^ Such mutations were identified in our investigation, as well.

There are 31 mutations in spike of BA.2 and 34 in BA.3, with 21 shared mutations among all.^22^ In March 2022, the BA.2 lineage was discovered in numerous nations, including Denmark and the United Kingdom. Even though BA.1 was the most prevalent variant during the Omicron wave of the pandemic, in many parts of the world, the number of cases of BA.2 has recently increased, indicating that BA.2 has a selective advantage over BA.1. It was thought that the greater capacity of BA.2 for immune escape was related to its growth advantage. However, the small difference in the level of neutralizing antibodies against BA.1 and BA.2 in those who received vaccination or who were infected with SARS‐CoV‐2 makes it difficult to explain why BA.2 is more transmissible than BA.1.^30^ Both BA.1 and BA.2 share several mutations, but each of them also have their own mutations. Due to the fact that the BA.2 S protein has more than 30 mutations in comparison to the original SARS‐CoV‐2 strain (Wuhan‐Hu‐1, GenBank: NC_045512.2), it is reasonable to wonder that the virological characteristics of BA.2 differ significantly from those of the original virus and the other variants.^31^, ^32^ The BA.2 lineage is identified by key S substitutions T19I, V213G, Δ25/27, L24S, T376A, and R408S.^32^ In our study, BA.2 showed several mutations in spike, such as ST376A, SR408S, and SV213G.

Omicron has 40 mutations including more than 30 mutations in protein S, one mutation in protein E, three mutations in protein M, and six mutations in protein N.^33^

In the Wuhan reference strain, an A‐to‐G nucleotide mutation occurred at position 23 403 and resulted in the spike D614G amino acid change, which was detected in early March 2020.^34^ Three additional mutations came along with the D614G substitution: A synonymous C‐to‐T mutation at position 3037, a nonsynonymous C‐to‐T mutation at position 14 408, and a C‐to‐T mutation at position 241 in the 5′ untranslated regions of the RNA‐dependent RNA polymerase gene.^35^ The monomeric S protein's affinity for ACE2 is unaffected by the mutation because residue 614 is outside of the receptor binding domain (RBD). Spike‐G614 was more effective than S‐D614 at ACE2‐mediated cell transduction by S‐pseudotyped vectors and at live SARS‐CoV‐2 infection of cells and animals.^36^ However, some studies suggested that the D614G mutation in the spike protein might make SARS‐CoV‐2 more infectious or more likely to spread and also can lead to the enhancement of viral replication and infectivity in human lung epithelial cells, which makes COVID‐19 more severe.^34^, ^37^, ^38^ Late in January 2020, the spike protein's D614G mutation was occasionally observed in both Europe and China. This mutation first spread to Europe and then gradually spread worldwide. It is still the predominant spike substitution, globally.^37^, ^39^ In our study, D614G mutation was continuously identified from the second wave to the sixth (with more than 70% frequency), and it was the major substitution. In the last wave, BA.1, BA.2, and mixed lineages (ML) showed this mutation at high percentage.

The N501Y mutation is localized to the RBD and helps in achieving higher binding affinity to host cells, potentially leading to increased transmission and infection. The SARS‐CoV‐2 N501Y.V1 (B.1.1.7) lineage has 17 nonsynonymous mutations and deletions, many of which are in the S protein. The N501Y.V2 (B.1.351) strain contains 10 mutations in the S protein, three of which are in the RBD (K417N, E484K, and N501Y). The N501Y.V1 and N501Y.V2 lineages exhibit higher transmission efficiency and immune evasion against neutralizing antibodies than the original strain.^40^, ^41^ In our investigation, N501Y was present during the fourth and sixth waves. But it was not detected in other waves.

P681H at the S1/S2 spike cleavage site is thought to increase furin cleavage, potentially affecting viral cell entry.^42^ It is believed that the mutations in Omicron's spike protein, P681H, increase spike protein cleavage and contribute to Omicron's high‐speed transmission.^43^ The presence of this substitution in Omicron raised concern as it may be associated with higher virulence and infectivity.^44^ In Iran, P681H was detected in the sixth and fourth waves. As in the last wave, this mutation was found in BA.1, BA.2, and mixed lineages with high frequencies.

Omicron has been described as a highly mutated variant with an “unusual constellation of mutations.”^45^ Free energy perturbation and computational mutagenesis could confirm that Omicron RBD binds ACE2, 2.5 times stronger than the prototype SARS‐CoV‐2. Notably, three substitutions, T478K, Q493K, and Q498R, nearly doubled the electrostatic potential (ELE) of the RBD^Omic^–ACE2 complex and made a significant contribution to the binding energies.^46^ Moreover, the Omicron variant and other VOCs share T478K and E484A mutations, which have been found to increase neutralizing antibody resistance and associate with immune escapes.^47^ In this study, T478K substitution was identified in the last two waves (fifth and sixth). In the sixth wave, this mutation was present in all BA.1, BA2, and mixed lineages samples.

The Omicron VOC is also described by the four‐point mutation in the Spike's N‐terminal domain: A67V, T95I, G142D, and N211I, with respective prevalence of 0.6, 24.8, 40.9, and less than 0.003%. Specifically, the in silico model has linked G142D to a change in the supersite epitope that binds NTD‐neutralizing antibodies.^48^ Like T478K, this mutation was detected in last two waves. Its frequency in BA.1, BA.2, and mixed lineages were more than 70%.

The G446S is another SARS‐CoV‐2 RBD's mutation in the Omicron BA.1 variant, which has an impact on how antigen is processed and presented and increases the antiviral activity of vaccine‐induced T cells. This makes it easier for T cells to recognize new variants.^25^ This mutation was not present in our study, in none of the waves.

We analyzed other structural genes including E, M, and N substitutions. About E gene, we detected the ET9I substitution in all BA.1, BA.2, and mixed lineages groups, which had more than 98% frequency. M‐A63T, M‐D3G, M‐Q19E, M‐G6S, and M‐A194V were observed in BA.1, BA.2, and mixed lineages for the M gene. M‐G6S was not detected in BA.2 and mixed lineages groups. About N gene mutations, E31del, G204R, P13L, R32del, A119T, R203K, PS33del, PG212S, P365L, and M101L were detected. N‐A119T was not presented in mix lineage group. N‐M101L was not identified in BA.2 and mixed groups.

It is important to note that nonstructural proteins of SARS‐CoV‐2 (NSPs) primarily affect the innate immune responses of humans, facilitating immune escape.

NSP3 has cleavage operations on nsp, via the pLpro domain, including self‐cleavage of NSP3.^49^ This nonstructural protein has several immune escape mechanisms that make it easier for viruses to reproduce, such as hindering ISG15 modification and inhibiting IFN production.^50^, ^51^, ^52^ In our study, NSP3 mutation was detected in BA.1, BA.2, and the mixed groups in the sixth wave, but not presented in previous waves.

The transmembrane proteins nsp3, nsp4, and nsp6 hijack and rearrange the membranes of the host endoplasmic reticulum, subsequently inducing the formation of double membrane vesicles (DMVs).^53^ We observed nsp4 substitution in the fourth, fifth, and sixth waves. But nsp6 mutations were just identified in the sixth wave in BA.1, BA.2, and mixed groups.

NSP5 is the major protease (Mpro) of the SARS‐CoV‐2. NSP5 likewise separates NLRP12 and TAB1 as well as handling long popular polypeptides. This protein is essential for viral infection.^54^ We observed NSP5 substitution just in the last wave in BA.1, BA.2, and mixed groups.

SARS‐CoV‐2 nsp12 besides its RdRp activities in viral replication blocks nuclear translocation of IRF3. Translocation of phosphorylated IRF3 into the nucleus is essential for IFN‐β transcription.^55^, ^56^, ^57^ This substitution was detected in all waves of COVID‐19 in Iran. In the sixth wave, we observed nsp12 mutation in BA.2 variants.

There are studies on accessory gene mutations and their impact on virus's cell cycle. Mutation in the Omicron variant's various accessory proteins, including ns3, ns6, ns7a, ns7b, and ns8 have been found.^58^ In our study, amino acid substitutions in accessory proteins were detected in a limited number of strains in the sixth wave. The rate of substitution in these proteins was among BA.1 and BA.2 variants as follows: 1.3% of BA.1 strains had NS7a‐P99S substitution and 0.6% of BA.2 variants contained NS7AE1STOP substitution. Besides, 1.3% of BA.2 strains had NS3‐H78Y substitution. NS3A72V was detected in 0.7% of BA.1 group. For ns6 region, NS6L35F and NS6I36T mutations found in BA.1 samples with 0.7% frequencies. The highest rate of mutations in accessory proteins belongs to NS7AT39I substitution with 5.6% in mixed lineage group. But ns8 substitution was detected in none of our samples.

In conclusion, we identified different lineages of SARS‐CoV‐2 Omicron variant (BA.1, BA.2, and mixed lineage) during the sixth wave and compared the substitutions in different SARS‐CoV‐2 genes in the sixth wave to previous five COVID‐19 waves in Iran. The results of this study showed that with the progression of the pandemic, the number of mutations increases significantly, which indicates an adaptive evolution of SARS‐CoV‐2 in humans to increase the transmission of the virus. To address this problem, it will be necessary to continuously monitor and analyze the effects of viral genome mutations to develop vaccines and antivirals that are effective against new SARS‐CoV‐2 variants.

## AUTHOR CONTRIBUTIONS

**Kaveh Sadeghi**: Performing tests. **Sevrin Zadheidar**: Performing tests. **Arghavan Zebardast**: Writing the manuscript. **Ahmad Nejati**: Investigation. **Marziyeh Faraji**: Data collection. **Nastaran Ghavami**: Data collection. **Shirin Kalantari**: Data collection. **Vahid Salimi**: Investigation. **Jila Yavarian**: Designing the study and editing the manuscript. **Adel Abedi**: Data analysis. **Nazanin Zahra Shafiei Jandaghi**: Data curation. **Talat Mokhtari‐Azad**: Supervision.

## CONFLICT OF INTEREST STATEMENT

The authors declare no conflicts of interest.

### PEER REVIEW

The peer review history for this article is available at https://www.webofscience.com/api/gateway/wos/peer-review/10.1111/irv.13135.

## Data Availability

Data are openly available in a public repository that issues datasets with DOIs.
